# BioInspired, BioDriven, BioMADE: The U.S. Bioindustrial Manufacturing and Design Ecosystem as a driver of the 4^th^ Industrial Revolution

**DOI:** 10.1049/enb2.12014

**Published:** 2021-09-13

**Authors:** Patrick P. Rose, Douglas Friedman

**Affiliations:** ^1^ Bioindustrial Manufacturing and Design Ecosystem US Office of Naval Research Global Ruislip UK; ^2^ Bioindustrial Manufacturing and Design Ecosystem or BioMADE Saint Paul Minnesota USA

**Keywords:** bio‐economy, biomaterials, industry, microbial engineering, scale‐up, strategies, synthetic biology, biotechnology, ecology, environmental factors, government, innovation management, Internet, manufacturing industries, organisational aspects, production engineering computing, research and development

## Abstract

When we think about the potential that biology has to offer, the U.S. Bioindustrial Manufacturing and Design Ecosystem or BioMADE slogan could read, ‘we don't make the products you buy, we make the products that you buy, with biology’. BioMADE is a non‐profit public–private partnership between the U.S. government and the private sector to leverage the work already accomplished in industry, accelerate the bioindustrial revolution, and create a stronger, resilient, sustainable, and environmentally friendly manufacturing ecosystem. BioMADE endeavours to be a leader, an enabler, and a beacon for how contemporary manufacturing can be transformed with biology to mature the bioindustrial manufacturing ecosystem. The institute cannot go this path alone to solve all the problems and coalesce the existing ecosystem. It requires determination and commitment from the private sector, academia, non‐profit research institutions and national laboratories; the entire community. Many technical challenges and adoption hurdles still loom high. Industry and consumers need to start accepting that engineering biology has a critical role to play in the manufacturing of many of the materials and products we use today.

## 1 INSPIRED BY BIOLOGY

The COVID‐19 pandemic has demonstrated how biology is a force to reckon with on our planet. Beyond infectious diseases, we often underestimate the design features of our planet's flora and fauna and mostly consider it frail and vulnerable. Yet, we have often observed or read about some fantastic features of plants or animals that allow them to flourish in their environment (e.g. the camouflage of the octopus, the power and efficiency of the bacterial flagellar motor, and the migratory bird's earth's magnetic field perception). Behind these remarkable designs are biological systems. Most of the past century has focussed on understanding how these biological systems function and, today, engineers harness them for the benefit of society. Scientists continuously seek to mimic or emulate these biological designs, primarily as a value added to agriculture and medicine. When we look beyond this horizon, we can see that the past decade has created new opportunities to leverage biological systems and create features and functions that touch practically every science and technology discipline (e.g. autonomous systems, information processing and storage, sense and sense making, and materials). The distinction of the more recent developments is the ability to harness the power of biology to create chemicals and materials that biology has not perfected to make.

With descriptors such as synthetic or engineering biology, which describes a long‐standing ability to engineer biology for a desired outcome, the distinction with the previous decades is our capability to use precision tools to manipulate biology (e.g. CRISPR). It has attracted a diverse array of scientists and engineers who recognize an opportunity to create something with ideal size, weight, and power metrics, to develop functions and features far superior to man‐made constructs. The inspiration comes from solving contemporary challenges and issues in power consumption, waste stream reduction, performance limitations, and sustainability, for example. Engineering biology allows for the integration of non‐natural technology and materials with biology to make hybrid products (e.g. emergent composite metamaterials and cybernetic interfaces) that have even better performance metrics. Physicists, chemists, aeronautical engineers, material and computer scientists, and architects work with biologists to apply contemporary non‐biological techniques (e.g. additive manufacturing, decision logic gates) to biology for predictive design and assembly of new materials. The anticipation is that we can take a phenomenal biological design, such as the flagellar motor (whose RPM exceeds 100,000; its motor is 100% electric; and energy to performance conversion is 100% efficient) to create new propulsion systems. The anticipated outcome of this convergence with biology is the long‐predicted Fourth Industrial Revolution, where manufacturing sources biology along with contemporary technology to build sustainable, secure, and resilient manufacturing processes that are environmentally friendly, sources waste products as feedstock, and creates a carbon net‐zero balance.

## 2 DRIVEN BY BIOLOGY

Biology is efficient. It has figured out how to maximise this efficiency in its conversion of feedstock to products. The process to get there is versatile and multifunctional. The processes are inherently redundant and adaptable. They are also energy efficient, can be used for on‐demand or continuous production, and can be highly cost effective. There are minimal waste streams and those that do exist are turned into feedstock within the production and processing ecosystem, thereby creating a nearly closed‐loop manufacturing structure. Biology has established how to convert different types of feedstock into products and leverage different types of production systems for optimal manufacturing. These attributes are desirable in any present‐day manufacturing ecosystem, and something every manufacturing industry aspires to achieve. The synthetic chemistry and petrochemical industry, for example, have evolved over a century to come close to acquiring many of those attributes that biology took many centuries to perfect. Other attributes have been difficult to obtain because the processes biology has created are nearly impossible to emulate or remain obscured by our lack of understanding. Again, take the flagellar motor—it is one of the most sophisticated, self‐assembled machines on earth and a target of recreation by many; it has yet to be achieved.

The key to getting behind the curtain and revealing the remaining unknowns in biology has been accelerated by advancements in engineering tools for harnessing biology. The last decade has been remarkable in science and technology advancements, which have given us many new and improved tools as well as novel insights into how we can translate such biological manufacturing efficiency to conventional industrial manufacturing for broad commercial consumption. Advances in machine learning, automation, rational design, and biological tools (e.g. CRISPR and DNA synthesis), for example, have created a makerspace that allows scientists to get a better hold on understanding biology. Even more exciting is the fact that we can learn from biology and improve biological processes. Biology has evolved over millennia to find an optimal conservation of energy and optimised production system, which is sometimes removed from a perfectly efficient system. What we can do today is improve the ‘good enough’ approach of nature into a near‐perfect system to create sustainable, ecologically friendly, resilient, and resource‐efficient manufacturing systems. The result is that many companies have successfully harnessed biology to improve or create new manufacturing processes. Others are looking to use biology to create sustainable and resilient production‐oriented supply chains and replace traditional production supplies that are costly, energy intensive, and not environmentally friendly.

## 3 BUILT WITH BIOLOGY: BioMADE

In the 1980s and 1990s, chemists were focussed on improving products through chemical manufacturing. Today, we think in terms of biology. When we think about the potential that biology has to offer, the U.S. Bioindustrial Manufacturing and Design Ecosystem or BioMADE slogan could read, ‘we don't make the products you buy, we make the products that make the products you buy, with biology’. Biomanufacturing has the capability to make fine chemicals, high‐value precursors, materials for additive manufacturing that can feed into contemporary synthetic chemistry and manufacturing at large.

Much of the work to date has been focussed on a proof‐of‐concept and modest scale; actual translation of these capabilities out of the laboratory and into full‐scale manufacturing has been lacking. This often has to do with underappreciating robust techno‐economic data to support actual cost, performance, and production metrics and to deliver superior products and reliable manufacturing capabilities. Outside biomedicine, for the past decade, government investment to build a non‐medical biomanufacturing industrial base has largely been unsuccessful; private investment does not have the incentives to provide the capital required. This will not change unless a more concerted effort is taken to shape the biomanufacturing industry. Meanwhile, the industries launched from these investments have mostly been unable to create value streams or establish themselves in profitable market sectors. Harnessing the power of engineering biology focussed on providing a service has shown to be successful in several instances.

The biomanufacturing industry's strength is in reducing waste streams to produce toxic intermediates and creating sustainable alternative feedstock to make rare or fine commodities, for example, that are otherwise limited in supply by geopolitical, environmental, geographic challenges, and sometimes even adverse market forces. At the same time, non‐biomanufacturing industries have been slow to recognize or adopt biomanufacturing as viable alternatives. There is a misconception that biology is soft, squishy, and vulnerable and has no place in manufacturing—there are plenty of examples to prove that wrong.

**FIGURE 1:**
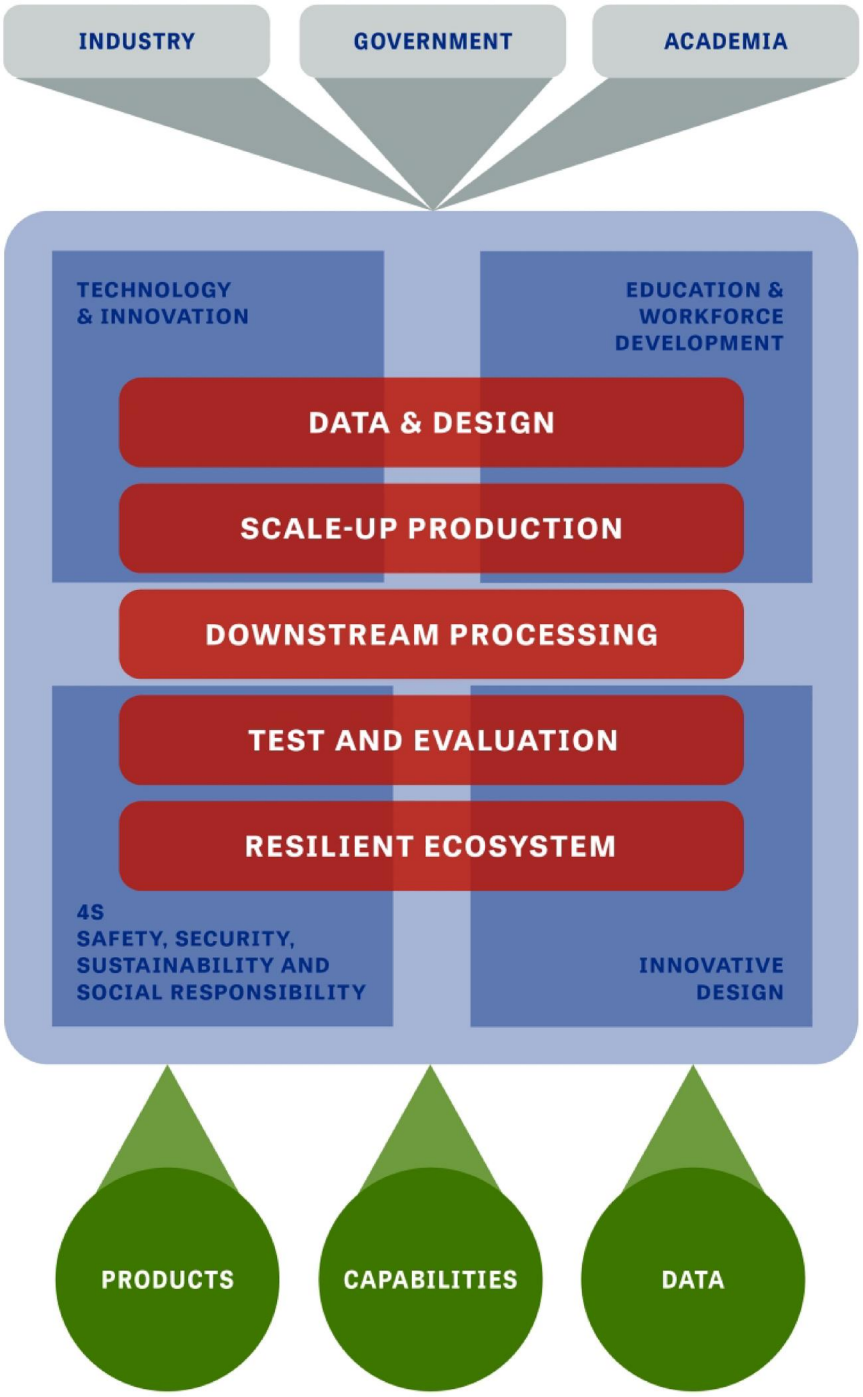
Overall vision of BioMADE. The Bioindustrial Manufacturing Institute advances biomanufacturing and supports the establishment and growth of supply chain intermediaries. BioMADE serves as a venue to bring together public and private entities in order to examine and advance industry‐wide standards, tools, and measurements, ELSI, biosecurity, and biosafety tenants for the responsible usage of this emerging technology

So why BioMADE, and why now? We are at an inflection point that other technologies have repeatedly gone through: take for example the many deaths and resurrections of artificial intelligence. Both industry and government recognise that a concerted effort can harness the mature technology and deliver on the promise of a *bio*industrial revolution. BioMADE is one focal point whose mission is to help make that happen (see Figure 1).

BioMADE is a non‐profit public–private partnership between the U.S. government and the private sector to leverage the work already accomplished in industry, accelerate the bioindustrial revolution, and create a stronger, resilient, sustainable, and environmentally friendly manufacturing ecosystem. It is a collection of members from across the ecosystem that come together to build a stronger foundation for bioindustrial manufacturing. BioMADE endeavours to be a leader, an enabler, and a beacon for how contemporary manufacturing can be transformed with biology to mature the bioindustrial manufacturing ecosystem. Biomanufacturing will not, and should not, replace the petrochemical or synthetic chemistry industry. BioMADE brings companies to the forefront who can make precursors, intermediate components etc., whose products eliminate waste streams, reduce energy consumption, replace the use of toxic industrial chemicals, and eradicate intensive processes that are not sustainable long‐term to provide a sustainable alternative. These precursors or intermediary products will be replacements into existing technologies and often with better performance, always made through a more economically and environmentally viable pathway. To achieve this lofty goal, there are four pillars of BioMADE: (1) technology development; to improve manufacturing tools through better downstream processes, improved chasses, scale‐up production, and data management, (2) workforce development; to provide viable entry points to enter the biomanufacturing workforce through lateral or vertical insertion, (3) addressing social, societal, safety, and security implications; to create a culture of responsibility, prosperity, and public understanding of the benefits of biomanufacturing, to ensure industry remains commercially viable by collaborating to adopt stringent security and safety protocols, and (4) innovative design; to encourage more risk taking to develop disruptive processes that create stepwise revolutionary changes in manufacturing.

The manufacturing innovation institute will address these four pillars with five thrust areas: (A) Data and Design; establish a unified computational infrastructure including a digital backbone and comprehensive datasets and include integrated approaches to the design‐build‐test‐learn cycle from proof‐of‐concept to on‐demand manufacturing, (B) Scale‐Up Production; develop tools for scale‐up biomanufacturing, including models and real‐time monitoring, and focus on rapid, predictable transition from lab‐scale to full‐scale production, (C) Downstream Processing; expand downstream processing capabilities to include novel recovery approaches and consider cell‐based downstream processing solutions to augment physical and chemical methods, (D) Test and Evaluation; enhance the ability to assess and characterize molecules, materials, and cells, and incorporate both cellular test and evaluation as well as molecule and performance metrics, and (E) Resilient Biomanufacturing Ecosystem; build an end‐to‐end domestic, distributed manufacturing supply chain.

We envision enabling biomanufacturing across the U.S., where local waste streams are sourced as feedstock and regional capabilities are integrated into the biomanufacturing hub. These manufacturers will create products to feed into regional existing manufacturing systems, no longer dependent on delivery of distant, sometimes rare or market‐driven products. No longer will we need enormously large manufacturing sites that are thousands of hectares large. Manufacturing will be more accessible and create opportunities for regional socio‐economic development because biomanufacturing hubs can go anywhere.

BioMADE's task is not easy. The Institute cannot go this path alone to solve all the problems and coalesce the existing ecosystem. It requires determination and commitment from the private sector, academia, non‐profit research institutions and national laboratories; the entire community. Many technical challenges and adoption hurdles still loom high. To date, one of the biggest challenges has been cost and time to effectively scale‐up production. It is currently too perilous to take one of the many proof of concepts and seamlessly introduce them into industrial manufacturing production. Better production capabilities need to be provided to ensure reliable production processes adaptable across multiple facilities, robust techno‐economic analyses must demonstrate market value, and performance metrics must communicate in standard terms how the product compares with existing standards.

Industry and consumers need to start accepting that engineering biology has a critical role to play in the manufacturing of many of the materials and products we use today. The institute will address design‐for manufacturing, scale‐up production, downstream processing, and test and evaluation to help companies get a better foothold in the market. BioMADE will also build a resilient ecosystem, focus on ethical, legal, and social implications, and advance education and workforce development. It will serve as a venue to bring together public and private entities in order to examine and advance industry‐wide standards, tools, and measurements, ELSI, biosecurity, and biosafety tenants for the responsible usage of this emerging technology. These products will include small molecule, biomineral, macromolecular, protein, and whole organism products to be used and/or further processed for a vast array of non‐medical applications. Specifically, BioMADE intends to develop capabilities and technologies for the production and scale of materials and processes from engineered biological systems.

BioMADE also does not pretend that it is the trailblazer in this technology area. Similar activities across the globe have been ongoing to raise the profile of biomanufacturing. Today, innovation is often handicapped by risk aversion and unwillingness to look forward. The community needs to rally around initiatives such as BioMADE, and BioMADE needs to rally with similar efforts across the globe to help transform the fabled 4^th^ Industrial Revolution into reality.

## CONFLICT OF INTEREST

The author declares to have no conflict of interest.

## PERMISSION TO REPRODUCE MATERIALS FROM OTHER SOURCES

None.

## Data Availability

Data sharing is not applicable − no new data generated.

